# Outcome observability in evidence on proton pump inhibitor-associated *clostridioides difficile* outcomes: a result-specific methodological audit

**DOI:** 10.3389/fmed.2026.1956355

**Published:** 2026-09-24

**Authors:** Jiawang Zhao, Ping Yin

**Affiliations:** Department of Pathophysiology, College of Basic Medical Science, Clinical Medical College of Anhui Medical University, Hefei, China

**Keywords:** clostridioides difficile infection, methodological heterogeneity, outcome observability, pharmacoepidemiology, proton pump inhibitors, methodological audit

## Abstract

**Objectives:**

Evidence on proton pump inhibitor (PPI) exposure and *Clostridioides difficile* infection (CDI) remains difficult to interpret because published estimates arise from heterogeneous target outcomes, risk sets, exposure contrasts, time structures, and ascertainment procedures. We evaluated whether a result-specific outcome-observability framework could make these differences explicit without using effect direction or statistical significance as classification criteria.

**Study design and setting:**

We conducted a structured methodological audit of evidence sources evaluating PPI exposure and CDI-related outcomes. Original observational reports and observational or randomized trial-based meta-analytic reports were retained as distinct evidence types.

**Methods:**

For each eligible reported result, we extracted the target outcome and risk set, PPI exposure contrast, time zero and exposure/outcome windows, outcome ascertainment, effect measure, event information, and data-source dependence. Four observability domains were coded as explicit/target-aligned (E), partial/proxy-based (P), unclear/misaligned (U), or aggregate/mixed (A). Codes were not summed, weighted, or dichotomized. Meta-analytic reports were described as aggregate evidence, and odds ratios, risk ratios, and hazard ratios were not treated as quantitatively interchangeable.

**Results:**

Eleven evidence sources contained an eligible PPI exposure contrast for a CDI-related outcome after one report was excluded during result-level reassessment. Eight addressed incident CDI, one addressed C. difficile detection without uniform confirmation of clinical CDI, one addressed severity among patients with established CDI, and one addressed mortality among patients with established CDI. Nine estimates were odds ratios, one was a risk ratio, and one was a hazard ratio. Temporal alignment and outcome ascertainment varied across original reports, while three meta-analytic reports combined heterogeneous component definitions. Two Swedish registry reports were treated as one overlapping data-source cluster.

**Conclusions:**

The included estimates did not represent one common PPI-CDI estimand. A result-specific outcome-observability profile can identify methodological non-comparability, but it is not a score of study quality, causal validity, statistical power, or likelihood of a positive result. The framework should be interpreted as a descriptive methodological audit rather than evidence that observability conditions explain the direction of reported associations.

## 1 Introduction

Proton pump inhibitors (PPIs) are among the most widely used medications for the prevention and treatment of acid-related gastrointestinal disorders. Their extensive and sometimes prolonged use has prompted sustained evaluation of potential adverse effects, including a possible association with *Clostridioides difficile* infection (CDI), a clinically important healthcare-associated infection associated with substantial morbidity and mortality (1).

Observational studies and meta-analyses have frequently reported positive associations between PPI exposure and CDI in adult hospital-based populations, population-based cohorts, and pediatric inpatient settings (2–6). In contrast, a meta-analysis restricted to randomized controlled trials reported a risk ratio whose confidence interval included the null (7). Pharmacovigilance evidence has additionally suggested that estimated associations may change after accounting for antibacterial co-exposure and competition bias (8). These findings have often been interpreted as a disagreement between observational and randomized evidence.

However, estimates labeled as PPI-CDI evidence do not necessarily address the same clinical question. Published reports differ in whether the target outcome is incident CDI, *C. difficile* detection, recurrent CDI, CDI severity among patients with established infection, or mortality after established CDI. They also differ in the population eligible to experience the outcome, the PPI comparator and exposure window, the index date or time zero, the duration of the outcome-risk period, and the procedure used to identify CDI. Consequently, apparently discordant estimates may represent different target estimands rather than alternative measurements of one common effect.

In this study, outcome observability refers to a result-specific description of whether the target outcome and eligible risk set, PPI exposure contrast, temporal sequence, and outcome-ascertainment procedure are sufficiently explicit to determine which clinical question an estimate addresses. This construct is narrower than study quality or causal validity and is distinct from statistical power. Statistical power depends on assumptions regarding effect size, event frequency, sample size, and the planned analysis, whereas outcome observability describes the exposure-outcome contrast that the reported study design and data were structured to represent. Dose and treatment duration may be clinically relevant attributes of PPI exposure (9, 10), but they do not by themselves establish causal validity or guarantee detection of an association.

This distinction is particularly relevant to drug-safety evidence. Non-statistically significant findings may be overinterpreted as evidence of no effect even when confidence intervals remain compatible with clinically important associations (11, 12). Sparse or rare adverse events can also limit inference from randomized trials and conventional meta-analytic methods (13). Conversely, observational and real-world data may provide complementary safety information when the data source is fit for the specific clinical question and relevant sources of confounding, selection bias, and measurement error are evaluated separately (14). Outcome observability therefore should not be used to privilege one study design over another or to infer that a particular result is causally correct.

Accordingly, this study did not aim to generate a pooled causal estimate of PPI-associated CDI risk or to determine whether observational or randomized evidence was more credible. Instead, we used PPI-CDI evidence as a model case to develop a result-specific outcome-observability framework and to examine whether published estimates addressed sufficiently comparable clinical questions. The framework describes differences in the target outcome and risk set, PPI exposure contrast, temporal alignment, and outcome ascertainment without using effect direction, effect magnitude, *P* values, or confidence-interval exclusion of the null as classification criteria.

## 2 Methods

### 2.1 Study design and unit of description

We conducted a structured methodological audit of published evidence concerning PPI exposure and CDI-related outcomes. The primary unit of description was the reported result rather than the publication as a whole. Before methodological profiling, each result was classified according to its target outcome and eligible risk set, including incident CDI, recurrent CDI, *C. difficile* detection, CDI severity among patients with established CDI, or mortality among patients with established CDI. Results representing different outcome strata were not treated as estimates of one common clinical outcome.

Original studies were described at the result level. Meta-analytic reports were retained as aggregate evidence sources but were not assigned a single study-level profile when their constituent studies differed in population, exposure definition, time origin, follow-up, or outcome ascertainment. Aggregate reports were used to describe the characteristics and limitations of the summarized evidence rather than as substitutes for their constituent primary studies.

### 2.2 Literature search and eligibility criteria

A systematic literature search was performed in PubMed, Web of Science, Embase (via Embase.com), and the Cochrane Library (CENTRAL) from inception through May 2026.

Reports were eligible when they evaluated a defined PPI exposure contrast for a CDI-related outcome and reported an extractable relative-effect estimate with uncertainty or sufficient data from which an effect estimate could be derived. Eligible evidence types included original observational studies, observational meta-analyses, and randomized trial-based meta-analyses.

Incident CDI, recurrent CDI, C. difficile detection without uniform confirmation of symptomatic infection, CDI severity among patients with established CDI, and mortality after established CDI were treated as separate outcome strata. A report was not considered an eligible PPI-CDI effect source merely because PPI use appeared as a participant characteristic or covariate. The modeled comparison had to estimate a defined PPI exposure contrast in relation to a subsequent CDI-related outcome.

### 2.3 Data extraction

Data were extracted using a structured result-level form. Extracted items included evidence-source type, study design, target outcome, eligible risk set, clinical setting, PPI exposure data source, comparator, reported effect measure and 95% confidence interval, index date or time zero, exposure look-back window or treatment-initiation date, outcome-risk period, follow-up and censoring, recurrence definition where applicable, CDI ascertainment procedure, sample size, event information, adjusted covariates, and potential overlap with other evidence sources.

For case-control studies, we recorded the CDI case date, the corresponding index date assigned to controls, and the pre-index PPI exposure window. For cohort and randomized evidence, we recorded treatment initiation or assignment, the start of outcome follow-up, the prespecified risk period, and censoring rules where available. For analyses of severity or mortality among patients with established CDI, CDI diagnosis was treated as cohort entry, and the temporal relationship between PPI exposure and the subsequent outcome was recorded separately.

Information was extracted as reported in the source article. When the index date, exposure window, follow-up period, censoring rule, or diagnostic procedure was not explicitly described, the item was classified as unclear rather than inferred.

### 2.4 Result-specific outcome-observability framework

Outcome observability was defined as a result-specific, multidimensional description of whether the reported target outcome and eligible risk set, PPI exposure contrast, temporal sequence, and outcome-ascertainment procedure were sufficiently explicit to determine which clinical question an estimate addressed. Outcome observability was not treated as a measure of statistical power, event frequency, study quality, risk of bias, causal validity, or the probability of obtaining a statistically significant result.

Four domains were evaluated: (1) target outcome and risk set, (2) PPI exposure contrast, (3) temporal alignment, and (4) outcome ascertainment. Each domain was described as explicit and target-aligned (E), partial or proxy-based (P), unclear or misaligned (U), or aggregate/mixed (A). The aggregate/mixed category was reserved for meta-analytic reports when heterogeneous constituent definitions could not be represented by a single study-level code.

The four domain codes were reported separately and were not summed, weighted, or converted into high- and low-observability categories. Baseline population risk, sample size, event count, precision, statistical power, study quality, and risk of bias were recorded separately and were not incorporated into the outcome-observability profile.

Domain assignments were based exclusively on reported design and measurement information. Effect direction, effect magnitude, *P* values, confidence-interval exclusion of the null, and the source authors' conclusions were not used as classification criteria. Each assignment was required to be supported by a study-specific description of the relevant methods, timing, or outcome definition.

Two reviewers (JZ and PY) independently evaluated each eligible result using the prespecified operational rules presented in Table 1. Domain assignments were based exclusively on reported design and measurement information. Effect direction, effect magnitude, *P* values, confidence-interval exclusion of the null, and the source authors' conclusions were not used as coding criteria. The original independent assignments were retained before consensus resolution. Disagreements were resolved through discussion and application of the prespecified domain rules. Inter-reviewer agreement was evaluated separately for each domain using raw percentage agreement and unweighted Cohen's kappa. Because the aggregate/mixed category was structurally applicable to meta-analytic reports, agreement was additionally evaluated after restriction to the eight original reports. Independent assignments, consensus classifications, and agreement statistics are presented in Supplementary Table S3.

**Table 1:** Operational rules for result-specific outcome-observability domains.

Domain	E—explicit/target-aligned	*P*—partial/proxy	U—unclear/misaligned	A—aggregate/mixed
Target outcome and risk set	Target outcome stratum, denominator, and eligible risk set are explicit and aligned with the reported result.	Outcome stratum is identifiable, but the denominator, risk set, recurrence status, or clinical state is incompletely specified.	The target outcome or eligible denominator cannot be determined from the report.	A meta-analytic report combines different target outcomes or risk sets across constituent studies.
PPI exposure contrast	PPI data source, comparator, and exposure window anchored to the index date/time zero are explicit; dose or duration is reported when relevant.	PPI use and comparator are identifiable, but dose, duration, adherence, or exposure window is incomplete.	The comparator or exposure window is not reported, or PPI exposure cannot be temporally anchored before the outcome.	Constituent studies use heterogeneous PPI definitions, comparators, or exposure windows.
Temporal alignment	Index date/time zero, exposure look-back or treatment start, outcome-risk period, and censoring are explicit; exposure precedes outcome.	Temporal precedence is apparent, but one or more elements of time zero, risk period, follow-up, or censoring are incomplete.	Exposure-to-outcome ordering is unclear, contemporaneous without sequence, or otherwise misaligned.	Constituent studies use heterogeneous time origins, exposure windows, follow-up periods, or censoring rules.
Outcome ascertainment	A prespecified clinical/laboratory rule or validated algorithm is explicit and appropriate for the target outcome.	An unvalidated administrative code, anti-CDI treatment proxy, laboratory positivity without compatible symptoms, or another partial proxy is used.	The outcome-ascertainment method is not sufficiently reported to determine how cases were identified.	Constituent studies use heterogeneous symptoms, tests, codes, treatment proxies, or adverse-event definitions.

Codes were treated as categorical descriptors and were not summed, weighted, or dichotomized. E, explicit and target-aligned; P, partial or proxy-based; U, unclear or misaligned; A, aggregate or mixed. The aggregate/mixed category was used only for meta-analytic reports when heterogeneous constituent definitions could not be represented by one study-level code.

### 2.5 Quality assessment

Methodological quality was assessed separately from the result-specific outcome-observability profile. The Newcastle-Ottawa Scale was applied to original observational studies, with the results reported in Supplementary Table S1. Meta-analytic reports were not assigned Newcastle-Ottawa Scale scores because they represented aggregated evidence rather than individual original studies. The randomized trial-based meta-analysis was described using the risk-of-bias information reported by its authors.

Quality assessment was used to describe potential limitations in study design and reporting. Newcastle-Ottawa Scale scores, risk-of-bias judgments, and overall study-quality assessments were not incorporated into any outcome-observability domain, were not used to modify domain codes, and were not combined with the four-domain profile.

Two reviewers (JZ and PY) independently assessed the eight original observational reports using the Newcastle–Ottawa Scale. Item-level disagreements were resolved through discussion, and consensus domain and total scores are reported in Supplementary Table S1.

### 2.6 Analytical approach

Evidence was summarized descriptively within prespecified target-outcome strata. Incident CDI, *C. difficile* detection, recurrent CDI, CDI severity among patients with established CDI, and mortality after established CDI were not treated as estimates of one common outcome. Original reports and meta-analytic reports were also described separately because aggregate estimates did not provide uniform study-level information on populations, exposure definitions, time origins, follow-up periods, or outcome ascertainment.

No pooled effect estimate, vote-counting analysis, or comparison of statistically positive vs. null findings was performed. Odds ratios, risk ratios, and hazard ratios were reproduced only in their originally reported metrics. These measures were not converted to a common effect measure or interpreted as quantitatively interchangeable because they represented different study designs, denominators, follow-up scales, comparators, and target outcomes. For descriptive visualization, the three effect-measure families were displayed in separate panels with panel-specific logarithmic axes.

Reports using overlapping populations or data sources were not interpreted as independent replications. In particular, the two Swedish registry reports covering substantially overlapping populations and calendar periods were treated as one data-source cluster during cross-study interpretation. Meta-analytic reports were considered dependent on their constituent studies and were not combined with primary reports in a study-counting analysis.

The previous threshold-based sensitivity analysis was not retained because the revised framework did not calculate a composite score or classify results as having high or low observability. Descriptive robustness summaries instead examined the effects of restricting interpretation to incident-CDI outcomes, original reports, results with explicit temporal ordering, and non-overlapping data-source clusters. These summaries were used to evaluate methodological comparability and were not interpreted according to whether effect estimates were statistically significant.

## 3 Results

### 3.1 Study selection

The literature search identified 13,840 records across four databases (PubMed, Web of Science, Embase, and Cochrane Library). After removal of 457 duplicates, 13,383 records were screened by title and abstract, of which 13,222 were excluded. The full texts of 161 articles were sought for retrieval; 86 could not be obtained. The remaining 75 full-text articles were assessed for eligibility. Of these, 41 were narrative reviews, editorials, comments, conference abstracts, or meta-analyses without extractable PPI–CDI effect estimates or sufficient methodological information; 3 were duplicate publications, 4 did not report CDI as a distinct outcome, and 15 provided no extractable effect estimate or 2 × 2 table data. A total of 11 evidence sources met the revised eligibility criteria and were included in the final methodological analysis, comprising eight original observational reports, two observational meta-analytic reports, and one randomized trial-based meta-analysis (Figure 1).

**Figure 1:**
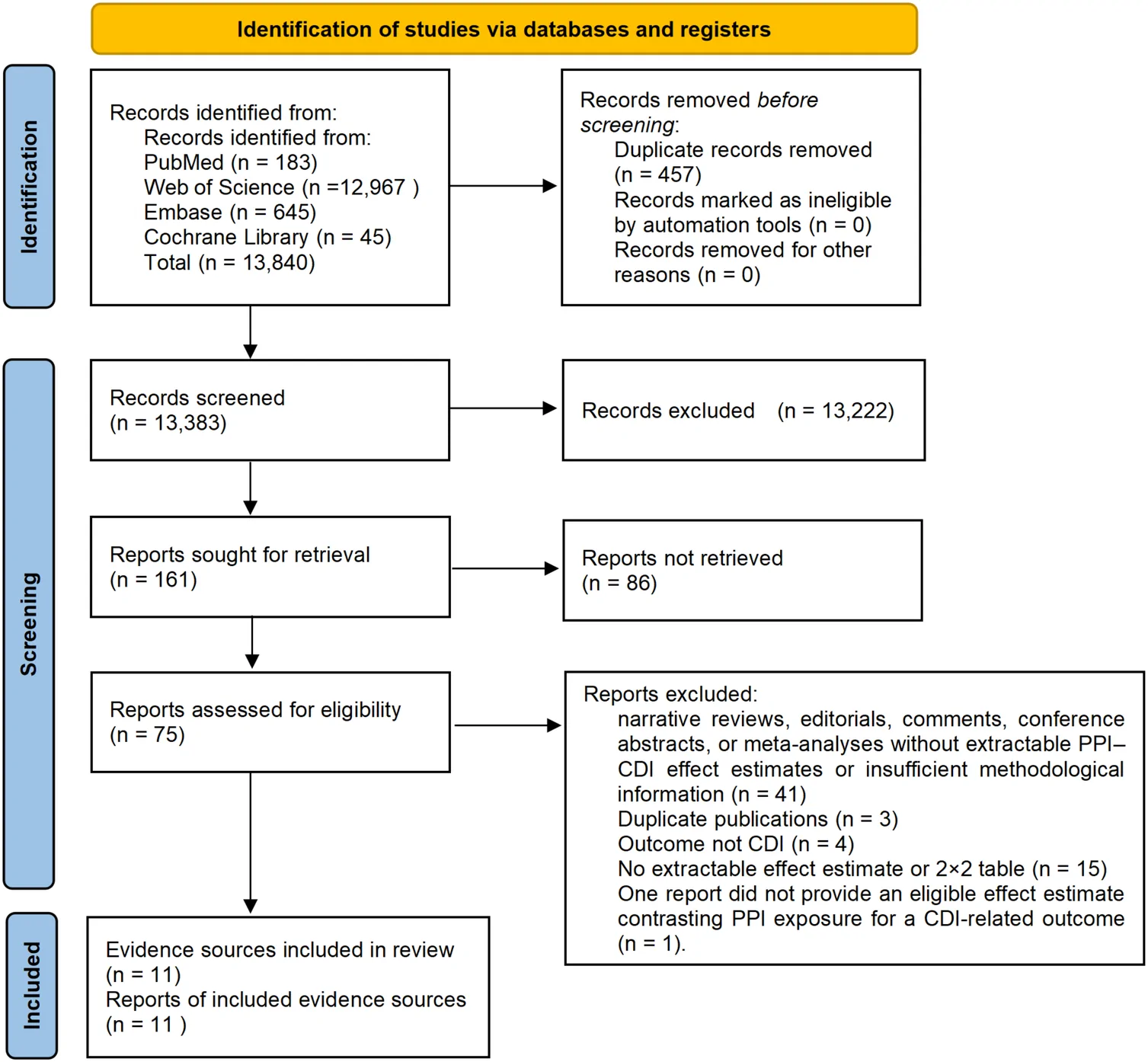
Flow diagram of evidence source identification, screening, eligibility assessment, and inclusion.

### 3.2 Characteristics of included evidence sources

We extracted the target outcome and eligible risk set, PPI comparator and exposure definition, index date or time zero, exposure and outcome windows, outcome-ascertainment procedure, event information, effect measure, and dependence between evidence sources. These characteristics were used to determine which clinical question each estimate addressed rather than to predict its direction or statistical significance (15–18).

The 11 eligible evidence sources comprised eight original observational reports, two observational meta-analyses, and one randomized trial-based meta-analysis. Eight reports addressed incident CDI, whereas one each addressed *C. difficile* detection, CDI severity among patients with established CDI, and mortality after established CDI. Nine estimates were reported as ORs, one as an RR, and one as an HR. Study populations ranged from selected inpatient groups to large population-based registry and electronic-health-record populations. Tables 2A, 2B show that the included estimates differed in target outcome, denominator, comparator, time origin, exposure window, follow-up structure, and outcome ascertainment.

**Table 2A:** Target estimands and effect measures of eligible PPI-CDI-related results.

Evidence source	Unit/design	Target outcome and risk set	PPI exposure comparison	Measure	Estimate (95% CI)	Direct-comparability note
Moreels (2)	Population-based case-control	First registry-coded CDI vs. population controls	Recent outpatient PPI dispensing vs. no corresponding dispensing	OR	2.65 (2.54–2.76)	Incident CDI estimate; recurrence is a separate outcome within the report.
Jiang (16)	Hospital-based observational/cross-sectional	C. difficile detection among diarrheal inpatients with pressure ulcers	PPI use vs. no PPI use	OR	6.95 (2.49–19.42)	Broader detection endpoint; only a subset was toxin positive.
Lin (20)	Cohort of established CDI	180-day all-cause mortality among patients with established CDI	PPI duration, modeled per additional week	HR	1.20 (1.01–1.43)	Post-CDI mortality, not incident CDI.
Boven (3)	Population-based case-control	Incident registry-coded CDI vs. population controls	Outpatient PPI dispensing within 30 days vs. no corresponding dispensing	OR	1.75 (1.68–1.82)	Highly overlapping Swedish data-source cluster with Moreels 2024.
Huang (9)	Nested case-control among new users	First treatment-defined CDI among new PPI or H2RA users	New PPI use vs. new H2RA use	OR	1.31 (1.22–1.40)	Active comparator; principal outcome used an anti-CDI treatment proxy.
Arriola (4)	Meta-analysis of observational studies	Hospital-acquired CDI across 23 observational studies	PPI exposure vs. non-exposure; definitions varied	OR	1.81 (1.52–2.14)	Aggregate result with substantial component heterogeneity (I2 = 82%).
Anjewierden (6)	Pediatric systematic review/meta-analysis	Pediatric inpatient CDI	PPI exposure vs. non-exposure; definitions varied	OR	1.33 (1.07–1.64)	PPI pooled estimate derived from four adjusted studies, not the full 14-study denominator.
Chang (18)	Pediatric CDI severity analysis	Severe vs. mild/moderate CDI among children with established CDI	PPI exposure in the prespecified pre-test month	OR	7.25 (1.07–49.07)	Severity among established CDI, not incident CDI.
Li (15)	Cross-sectional inpatient study	CDI among IBD inpatients with diarrhea	PPI use within the prior month vs. no use	OR	2.39 (1.07–5.35)	43 CDI events; wide confidence interval.
Boustany (5)	Retrospective multi-institution EHR study	Inpatient CDI diagnosis vs. no diagnosis	Inpatient PPI medication order vs. no corresponding order	OR	3.27 (3.23–3.30)	PPI was a secondary covariate; exposure-to-outcome ordering was not reported.
Floria (7)	Meta-analysis of 8 placebo-controlled RCTs	CDI adverse events	PPI vs. placebo	RR	1.19 (0.75–1.89)	CDI was not a primary trial outcome; diagnostic methods and follow-up varied.

Effect measures are reproduced in their originally reported metrics; magnitudes are not interpreted as directly comparable across different outcomes, risk sets, comparators, or time scales. Suljagić et al. (17). was excluded because the reported estimate did not represent an eligible PPI-exposure contrast for a CDI-related outcome.

**Table 2B:** PPI exposure definition, temporal alignment, outcome ascertainment, and event information.

Evidence source	PPI contrast/definition	Time origin and exposure/risk window	Outcome ascertainment	Sample/event information	Key uncertainty or dependency
Moreels (2)	At least one outpatient dispensing; recent 0–30 days and preceding 31–180 days	First CDI date/control proxy index; pre-index look-back windows	Swedish registry ICD-10 A04.7	43,152 cases; 355,172 controls; 17% recurrence within 8 weeks	Administrative code not reported as a validated clinical phenotype; overlaps Boven 2026 data source.
Jiang (16)	PPI use yes/no; no reliable pre-specimen duration/window reported	Fresh stool collected during admission; exposure-to-specimen sequence unclear	Diarrheal inpatients; culture/GDH positivity; toxin A/B in a subset	120 patients; 39 culture/GDH positive; 12 toxin positive	Do not label all 39 as uniformly toxin-confirmed clinical CDI.
Lin (20)	Recent daily PPI use; duration modeled per week	CDI diagnosis/cohort entry; mortality assessed through 180 days	Diarrhea plus tcdB PCR for CDI cohort; all-cause death as outcome	240 CDI patients; 91 deaths within 180 days	Post-CDI mortality estimand; PPI exposure timing/handling is only partly specified.
Boven (3)	Outpatient PPI prescription within 30 days	Case CDI date/control index; 30-day pre-index window	Swedish registry ICD-10 A04.7	42,921 cases; 355,159 controls	Administrative code; highly overlapping Swedish 2006–2019 cluster with Moreels 2024.
Huang (9)	New PPI vs. new H2RA use; as-treated duration	First prescription = time zero; censor at discontinuation, switch, death, CDI, discharge, or study end	Oral metronidazole/fidaxomicin/vancomycin for >=7 days; stricter sensitivity definition	6,933 cases; 6,933 controls	Principal outcome is a treatment proxy, although a stricter sensitivity algorithm was evaluated.
Arriola (4)	At diagnosis, during index admission, or within prior 90 days; varied by component	Constituent look-back/follow-up periods varied	10 studies symptoms + toxin; 11 toxin without documented symptoms; 2 ICD-9	23 studies; 186,033 participants; 10,307 CDI; I2 = 82%	Aggregate report; no single study-level profile is defensible.
Anjewierden (6)	PPI definitions varied; pooled result used adjusted estimates	Mixed or not consistently reported across components	Toxin, culture/toxin, or ICD/billing methods across components	14 studies overall; 10,531,669 children; 22,320 CDI; PPI estimate from 4 adjusted studies	Do not attribute the full pediatric denominator to the four-study PPI contrast.
Chang (18)	Medication exposure from 1 month to 48 h before positive test	First positive test/CDI episode; severity among established CDI	Diarrhea plus toxigenic PCR; explicit severe-complicated rule	49 CDI cases: 13 severe; 36 mild/moderate	Severity estimand, not incident CDI.
Li (15)	PPI use within prior month	Admission/stool diagnosis; pre-diagnosis exposure window	Diarrhea plus GDH/toxin algorithm with NAAT arbitration; culture	317 IBD inpatients with diarrhea; 43 CDI	Small event count and wide confidence interval.
Boustany (5)	EHR PPI drug order mapped by RxNorm	One-year CDI incidence reported; exposure-to-CDI sequence not specified	Inpatient SNOMED diagnosis; phenotype validation not reported	55,664,420 hospitalized adults; 48,720 CDI	Total population is not a matched control count; temporal ordering is unclear.
Floria (7)	PPI vs. placebo; regimens varied across 8 RCTs	Follow-up ranged from hospital stay/days to approximately 3 years	CDI was not a primary outcome; diagnostic methods varied	29,880 participants; 60/14,946 vs. 49/14,934 CDI events	Aggregate trial evidence with heterogeneous follow-up and ascertainment.

Cells report source-level facts. Missing timing or diagnostic information is labeled unclear rather than inferred. Cells reproduce source-level methodological information. Timing, exposure, follow-up, or diagnostic information not explicitly reported in the source article was classified as unclear rather than inferred. Event information and precision were recorded separately and were not incorporated into the outcome-observability profile.

### 3.3 Result-specific outcome-observability profiles

Among the original reports, the target outcome and eligible risk set were generally identifiable, but the PPI exposure window, exposure-to-outcome ordering, or outcome phenotype was incomplete in several sources. Administrative-code, treatment-based, and laboratory-only proxies were distinguished from explicit clinical or multistep laboratory definitions. Missing information was described as unclear rather than interpreted as evidence that the corresponding method had not been implemented.

The three meta-analytic reports combined constituent studies with heterogeneous populations, exposure definitions, time origins, follow-up periods, or outcome-ascertainment procedures. These reports were therefore described using the aggregate/mixed category where appropriate and were not assigned a single publication-level observability score. The consensus result-specific outcome-observability profiles for all 11 evidence sources are presented in Table 3.

**Table 3:** Result-specific outcome-observability profiles without a composite score.

Evidence source	Target outcome/risk set	PPI exposure contrast	Temporal alignment	Outcome ascertainment	Dependency/key interpretation
Moreels (2)	E—first registry CDI vs. population controls	E—dispensing in two pre-index windows	E—case date/control proxy date explicit	P—unvalidated administrative code	Same Swedish 2006–2019 data-source cluster as Boven 2026.
Jiang (16)	P—C. difficile detection in diarrheal pressure-ulcer inpatients	U—PPI use lacks a reliable reported pre-specimen window	U—exposure-to-specimen sequence unclear	P—culture/GDH positive; only 12 toxin positive	Do not label the endpoint as uniformly toxin-confirmed clinical CDI.
Lin (20)	E—180-day mortality among established CDI	P—daily use/duration; timing only partly specified	P—CDI entry and mortality window explicit; exposure handling partial	E—defined CDI cohort and death outcome	Different risk set and estimand from CDI occurrence.
Boven (3)	E—incident registry CDI vs. population controls	E—dispensing within 30 days	E—case/control index window explicit	P—unvalidated administrative code	Same Swedish 2006–2019 data-source cluster as Moreels 2024.
Huang (9)	E—first treatment-defined CDI among new users	E—PPI vs. H2RA from first fill	E—as-treated start and censoring explicit	P—anti-CDI treatment proxy; stricter sensitivity rule	Active comparator; not PPI vs. nonuse.
Arriola (4)	A—23 heterogeneous hospital studies	A—at diagnosis/index admission/prior 90 days	A—mixed component windows	A—symptoms + toxin, toxin-only, or ICD-9	I2 = 82%; no single study-level profile.
Anjewierden (6)	A—pediatric inpatient studies	A—PPI definitions mixed	A—mixed/not consistently reported	A—toxin, culture, and administrative codes	PPI pooled estimate derives from four adjusted studies.
Chang (18)	E—severe disease among established pediatric CDI	E—use in prespecified prior-month window	E—pre-test exposure window explicit	E—clinical/laboratory CDI and severity rule	Not an incident-CDI result.
Li (15)	E—CDI among IBD inpatients with diarrhea	E—PPI use within prior month	E—pre-diagnosis window explicit	E—symptoms plus multistep laboratory algorithm	43 events; wide confidence interval.
Boustany (5)	E—inpatient CDI diagnosis vs. no diagnosis	U—PPI order without temporal anchoring	U—exposure-to-outcome ordering unclear	P—SNOMED diagnosis; validation not reported	PPI was a secondary covariate.
Floria (7)	A—CDI adverse events in 8 RCTs	A—PPI vs. placebo regimens varied	A—follow-up from days/hospital stay to about 3 years	A—non-primary outcome; variable diagnostic methods	60 vs. 49 events; component-level audit needed.

Effect estimates were omitted from the profiling table because effect direction, magnitude, and statistical significance were not coding criteria. Domain codes are reported separately and are not summed, weighted, or dichotomized. Aggregate/mixed codes indicate that heterogeneous constituent definitions could not be represented by one study-level code.

Across the 44 independent domain assignments, the two reviewers agreed on 36 assignments, corresponding to an overall raw agreement of 81.8%. Domain-specific raw agreement was 90.9% for the target outcome and risk set, 81.8% for the PPI exposure contrast, 63.6% for temporal alignment, and 90.9% for outcome ascertainment; the corresponding unweighted Cohen's kappa values were 0.81, 0.73, 0.48, and 0.86, respectively. After restriction to the eight original reports, overall raw agreement was 87.5% (28/32 assignments). Temporal alignment showed the lowest agreement (62.5%; *κ* = 0.37), whereas target outcome/risk set and outcome ascertainment each showed 100% agreement (*κ* = 1.00) (Supplementary Table S3).

### 3.4 Differences in estimands, temporal alignment, and evidence aggregation

The included estimates did not represent a single common PPI-CDI outcome. Eight reports evaluated incident CDI, whereas Jiang et al. (16). evaluated C. difficile detection, Chang et al. (18). evaluated CDI severity among patients with established CDI, and Lin et al. (20). evaluated mortality after established CDI. These outcome strata had different eligible denominators and time origins and were therefore summarized separately. Among the incident-CDI reports, Li et al. (15) evaluated CDI among inpatients with inflammatory bowel disease and diarrhea.

Temporal definitions also varied substantially. Some case-control reports assessed outpatient PPI dispensing within a prespecified period before the CDI case date or the corresponding control index date. Huang et al. used the first PPI or H2-receptor-antagonist prescription as time zero and applied an as-treated follow-up structure. In contrast, the temporal ordering of PPI exposure and CDI diagnosis was not clearly specified in some cross-sectional or electronic-health-record analyses. Meta-analytic reports combined heterogeneous exposure windows, follow-up periods, and outcome definitions and were therefore described as aggregate/mixed rather than assigned one study-level profile.

Moreels et al. and Boven et al. used substantially overlapping Swedish registry populations and calendar periods from 2006 to 2019. These reports were treated as one data-source cluster rather than as independent replications. In addition, the effect estimate reported by Suljagić et al. was not interpreted as a PPI-to-CDI effect estimate because all participants already had healthcare-associated CDI and the modeled comparison concerned medical vs. surgical cases.

Reported estimates are displayed descriptively in Figure 2 according to effect-measure family. ORs, the RR, and the HR are shown in separate panels with panel-specific logarithmic scales because they represent different estimands, denominators, study designs, and time scales. No pooled estimate or comparison of statistically positive vs. null findings was performed.

**Figure 2:**
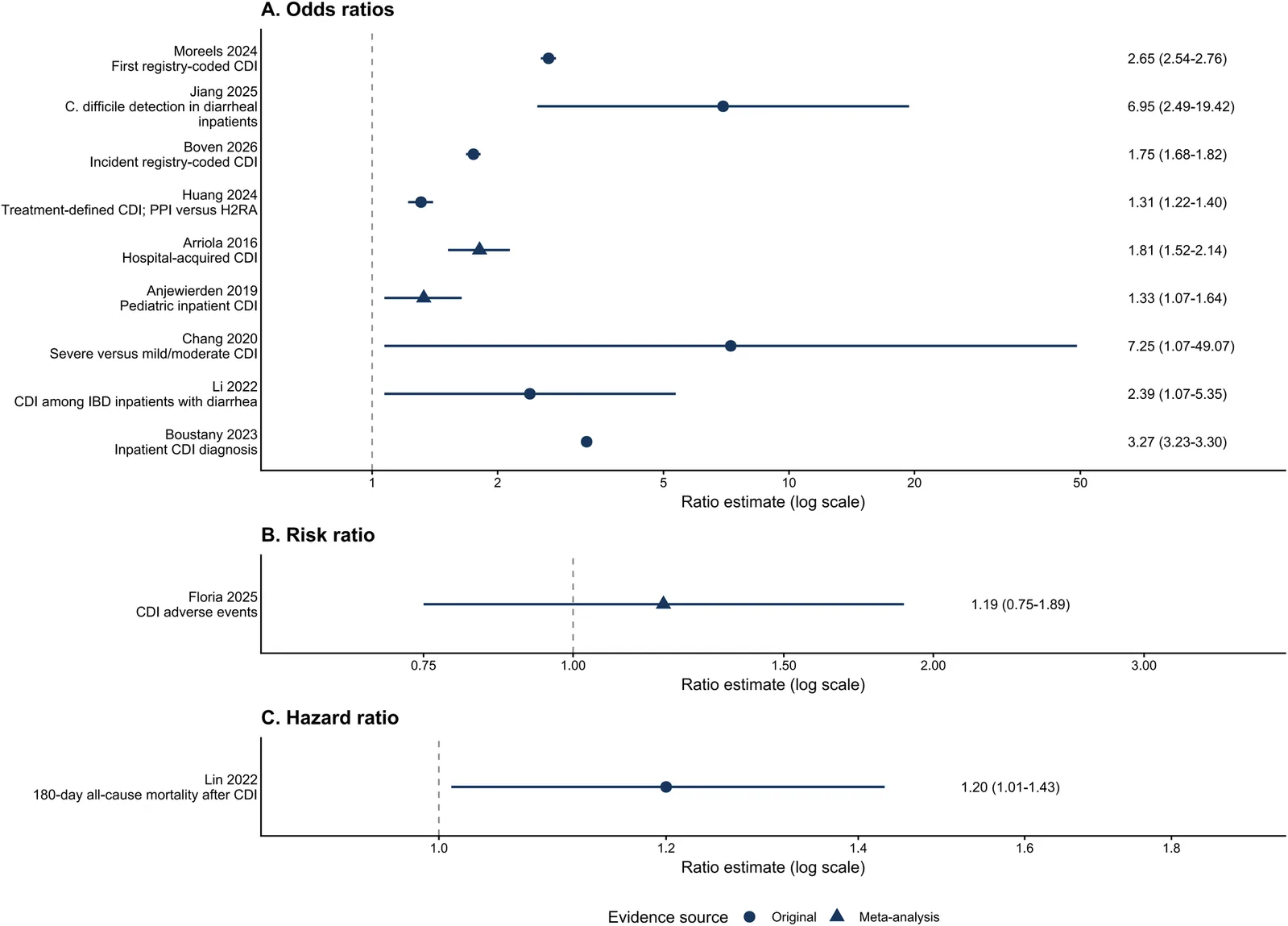
Reported PPI-CDI-related effect estimates grouped by effect-measure family: **(A)** odds ratios; **(B)** risk ratio; and **(C)** hazard ratio. Effect estimates are displayed in separate panels using panel-specific logarithmic scales. The dashed line indicates the null ratio of 1. Estimates are reproduced in their originally reported metrics and should not be directly compared across panels or across different target outcomes, risk sets, comparators, or time scales. Circles represent original reports, and triangles represent meta-analytic reports.

### 3.5 Conceptual synthesis

Figure 3 summarizes the revised result-specific outcome-observability framework. The framework begins by defining the target clinical question, including the outcome stratum and eligible risk set, and then describes the PPI exposure contrast, temporal alignment, and outcome-ascertainment procedure. The resulting E/P/U/A profile is used to assess whether published estimates address sufficiently comparable clinical questions. Event count, precision, statistical power, study quality, risk of bias, effect direction, and statistical significance are evaluated separately and are not incorporated into the profile.

**Figure 3:**
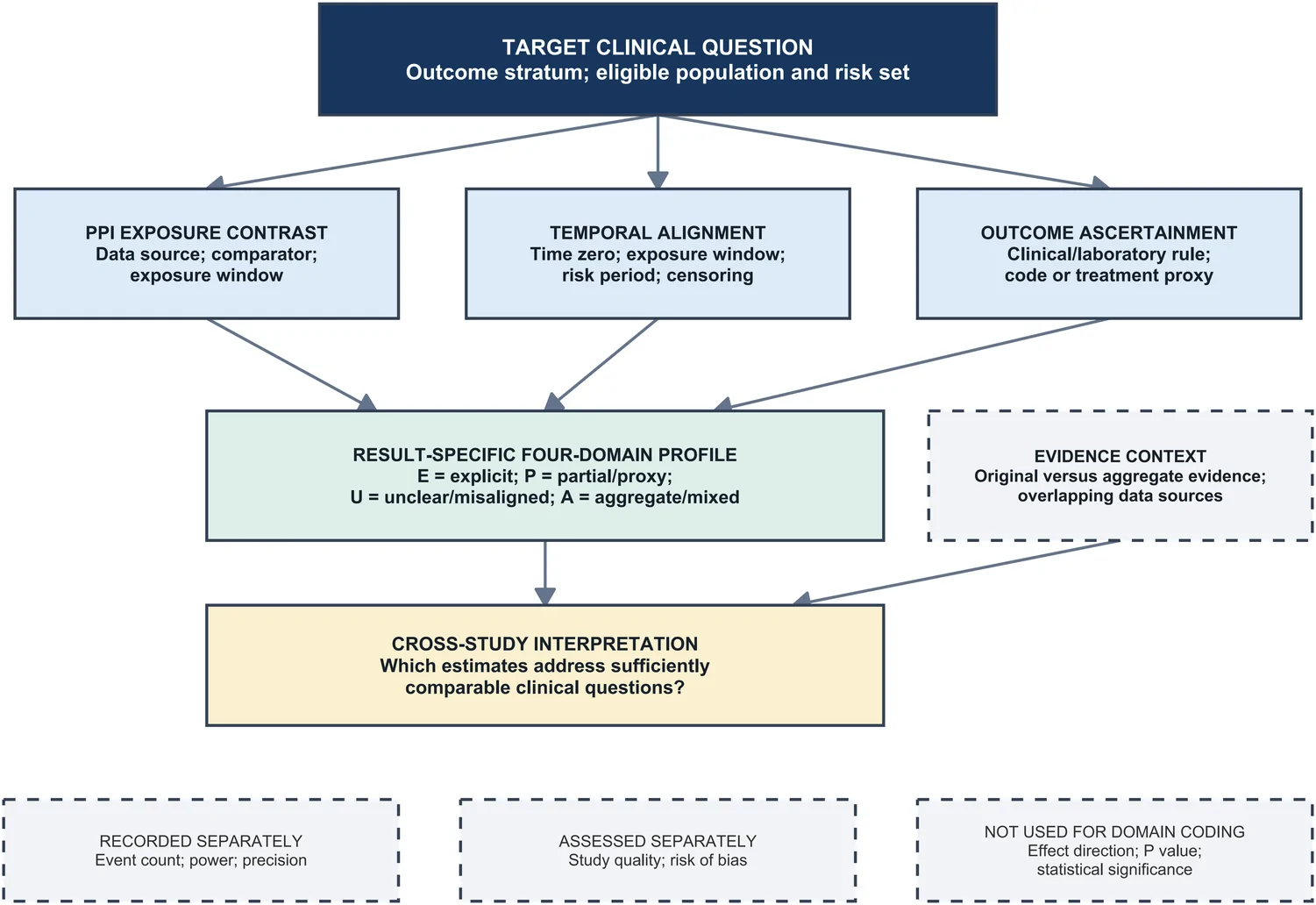
Result-specific outcome-observability framework. The framework first identifies the target clinical question and then describes the PPI exposure contrast, temporal alignment, and outcome ascertainment for each reported result. Domain codes are reported separately and are used to evaluate whether published estimates address sufficiently comparable clinical questions. Evidence aggregation and data-source dependence are considered during interpretation. Event count, precision, statistical power, study quality, risk of bias, effect direction, and statistical significance are evaluated separately and are not incorporated into a composite observability score.

## 4 Discussion

### 4.1 Principal findings

This methodological audit identified differences more fundamental than a high-vs.-low detectability classification. The included reports evaluated non-equivalent outcome strata, used different PPI exposure contrasts and time origins, applied heterogeneous CDI-ascertainment procedures, and included both original and aggregate evidence. Two large registry reports also represented substantially overlapping data-source populations rather than independent replications.

These differences determined which clinical question each reported estimate addressed and substantially limited direct cross-study comparison. They did not establish whether a particular estimate was causally valid, whether the underlying PPI-CDI association was null or non-null, or whether outcome-observability conditions explained the direction or statistical significance of the reported findings. The revised framework therefore functions as a descriptive methodological audit rather than an explanatory model of discordant effect estimates.

### 4.2 Outcome observability vs. power, precision, and study quality

Outcome observability should be distinguished from statistical power, precision, study quality, and risk of bias. Statistical power is the probability of rejecting a null hypothesis under specified assumptions regarding effect size, event frequency, sample size, and analysis. Precision is reflected by the uncertainty surrounding an estimate. Study quality and risk of bias concern internal validity, including confounding, selection, exposure misclassification, outcome misclassification, and analytical limitations. In contrast, outcome observability describes whether the reported exposure-outcome structure is sufficiently explicit to determine which clinical question an estimate addresses.

These dimensions may diverge. A result may have a clearly specified PPI contrast, time origin, risk period, and outcome definition but remain vulnerable to confounding or selection bias. Conversely, a carefully designed study may produce an imprecise estimate because few CDI events occurred. Non-statistically significant findings should therefore be interpreted using their confidence intervals, event information, and target estimand rather than automatically treated as evidence of no effect (11, 12, 19). Similar interpretive difficulties arise when adverse events are rare or were not primary trial outcomes (13, 21).

### 4.3 Implications for interpretation and evidence synthesis

The principal interpretive implication is that study-design labels alone do not establish that two estimates answer the same safety question. Before comparing observational and randomized evidence, investigators should identify the eligible risk set, target outcome, comparator, exposure window, time zero, outcome-risk period, and ascertainment procedure. Randomized and real-world evidence may provide complementary safety information when each data source is fit for the specific question and its precision and potential biases are evaluated separately (14).

Positive observational associations should not be accepted solely because their exposure and outcome definitions appear explicit. Evidence that estimated PPI-CDI associations may be attenuated after accounting for antibacterial co-exposure and competition bias illustrates the continuing importance of confounding structure and data-source limitations (8). Similarly, a randomized estimate whose confidence interval includes the null should not automatically be interpreted as proving the absence of risk. Outcome observability does not resolve either problem; it clarifies the question to which each estimate applies.

For evidence synthesis, non-equivalent outcome strata and effect measures should not be combined merely because they concern PPI exposure and a CDI-related label. ORs, RRs, and HRs retain different interpretations and should not be treated as directly interchangeable. When a study-level methodological profile is required, meta-analytic reports should be examined through their constituent primary studies. If component-level extraction is not performed, heterogeneous exposure, timing, and ascertainment definitions should remain explicitly classified as aggregate/mixed. Overlapping registries and overlapping meta-analytic components should also be recognized before apparent replication is interpreted.

### 4.4 Circularity and framework interpretation

Circularity would arise if characteristics expected to increase the probability of signal detection were used to classify studies as having high detectability and the greater frequency of statistically positive findings in that group were then presented as validation of the framework. To avoid this problem, the revised framework removed the composite score, high/low classification, threshold analyses, and positive-vs.-null comparisons. The four domains were defined using reported design and measurement information only, and effect direction, magnitude, *P* values, and confidence-interval exclusion of the null were not coding criteria (19).

The framework should therefore not be interpreted as a validated instrument for predicting whether a study will produce a positive result. Future validation should examine inter-reviewer reliability, content validity, and reproducibility in an external evidence corpus or in simulations with a known data-generating mechanism. Association with statistical significance in the same corpus would not constitute adequate validation (21).

### 4.5 Strengths and limitations

A principal strength of this study is the shift from asking whether a study was structurally capable of detecting an association to asking which clinical question each reported estimate addressed and whether that question was comparable across reports. Additional strengths include result-level profiling, explicit separation of outcome strata and effect measures, design-specific recording of time zero and exposure windows, recognition of aggregate evidence and overlapping data sources, and exclusion of effect direction and statistical significance from the coding rules.

Several limitations should be acknowledged. First, domain classification requires judgment, and the framework has not undergone formal content-validity or external validation. Second, the evidence corpus was small, particularly for randomized evidence. Third, meta-analytic reports could not be characterized at the original-study level without re-extracting every constituent trial or observational study. Fourth, incomplete reporting was classified as unclear, but the audit could not determine whether an incompletely reported procedure had nevertheless been implemented appropriately. Fifth, the outcome-observability profile does not control confounding, selection bias, measurement error, or other threats to causal inference. Finally, this study was not designed to estimate the causal magnitude of PPI-associated CDI risk, and its findings should not be interpreted as demonstrating that methodological observability explains discordant effect estimates.

Inter-reviewer agreement was lower for temporal alignment than for the other domains, indicating that classification of incompletely reported time zero, exposure windows, and outcome-risk periods remains an area requiring further refinement and external validation.

## 5 Conclusions

Outcome observability can be retained as a result-specific methodological framework for describing whether published PPI-CDI estimates address comparable clinical questions. The present audit identified differences in target outcomes, eligible risk sets, exposure contrasts, temporal alignment, outcome ascertainment, evidence aggregation, and data-source dependence. These differences limited direct cross-study comparison but did not demonstrate that observability conditions explained the direction or statistical significance of the reported estimates. The framework should therefore be interpreted as a descriptive methodological audit rather than a validated scoring instrument, a predictor of positive findings, or a basis for causal inference.
