# Supplementary material for: Outcome observability in evidence on proton pump inhibitor-associated clostridioides difficile outcomes: a result-specific methodological audit
Source: Front Med (Lausanne). 2026 Sep 24;13:1956355. doi: 10.3389/fmed.2026.1956355 (PMC13646726; doi:10.3389/fmed.2026.1956355)
Supplement: Supplementary file 1 [file Supplementaryfile1.docx]

**Supplementary Table S1. Newcastle-Ottawa Scale assessment of retained original observational reports.**

Two reviewers (JZ and PY) independently assessed the eight original observational reports using the Newcastle–Ottawa Scale. The original item-level ratings were compared after both assessments had been completed. Disagreements were resolved through discussion, and the consensus domain and total scores are reported in Supplementary Table S1. These ratings were used only for the descriptive NOS ≥7 restriction and were not incorporated into the outcome-observability classifications.

| **Study** | **Selection (0-4)** | **Comparability (0-2)** | **Outcome/Exposure (0-3)** | **Total (0-9)** |
| --- | --- | --- | --- | --- |
| Moreels 2024 | 4 | 2 | 2 | 8 |
| Jiang 2025 | 3 | 2 | 2 | 7 |
| Lin 2022 | 4 | 2 | 2 | 8 |
| Boven 2026 | 4 | 2 | 2 | 8 |
| Huang 2024 | 3 | 2 | 2 | 7 |
| Chang 2020 | 3 | 1 | 2 | 6 |
| Li 2022 | 3 | 1 | 2 | 6 |
| Boustany 2023 | 3 | 2 | 2 | 7 |

Note: For cohort studies, the third NOS domain referred to outcome assessment; for case-control studies, it referred to exposure ascertainment. NOS, Newcastle-Ottawa Scale.

**Supplementary Table S2. Eligibility corrections, evidence-source dependencies, and rule-based robustness analyses.**

To examine robustness without using statistical significance to define classifications, we applied rule-based methodological restrictions specified without reference to the direction, magnitude, confidence interval, or P value of each result. These post hoc analyses were descriptive and were not intended to validate the outcome-observability framework.

| **Audit or robustness item** | **Sources affected** | **Rule** | **Resulting evidence set** | **Effect on interpretation** |
| --- | --- | --- | --- | --- |
| Eligibility correction | Suljagic 2020 | Exclude the reported medical-versus-surgical CDI-case OR from the PPI-CDI evidence summary. | 11 eligible report-level effect summaries. | Removed one incorrectly directed and non-eligible OR; the eligible evidence base decreased from 12 to 11 report-level effect summaries. |
| Data-source dependence | Moreels 2024; Boven 2026 | Treat the two reports as one Swedish 2006-2019 registry cluster when considering apparent replication. | 10 report-level evidence clusters. | Moreels 2024 and Boven 2026 were not treated as independent replication, reducing 11 reports to 10 evidence clusters. |
| CDI-occurrence focus | Jiang 2025; Lin 2022; Chang 2020 excluded | Remove broader C. difficile detection, post-CDI mortality, and CDI-severity strata from the CDI-occurrence subset. | 8 reports directly addressing CDI occurrence. | Cross-outcome estimates were not treated as directly comparable CDI-incidence outcomes. |
| Primary-study focus | Arriola 2016; Anjewierden 2019; Floria 2025 excluded | Restrict study-level classification to original observational reports. | 8 original observational reports. | The three meta-analytic sources remained aggregate/mixed evidence and did not receive uniform study-level classifications. |
| Explicit temporal ordering | Jiang 2025 and Boustany 2023 excluded; aggregate reports addressed by component audit | Require exposure assessment before a defined index date or time zero. | Moreels 2024, Lin 2022, Boven 2026, Huang 2024, Chang 2020, and Li 2022 remained; Lin 2022 was classified as partial. | Eligibility was determined by temporal anchoring rather than statistical significance, demonstrating that temporal alignment was not uniformly explicit. |
| Clinical/laboratory ascertainment | Li 2022 and Chang 2020 most explicit; Lin 2022 explicit for its CDI cohort | Require compatible symptoms plus toxigenic/toxin evidence and match any secondary outcome to its risk set. | No sufficiently homogeneous incident-CDI subset was identified across all designs. | No pooled quantitative sensitivity analysis was attempted because codes, treatment proxies, organism detection, and adverse-event endpoints were not equivalent. |
| Arriola component audit | 23 observational studies | Retain the aggregate/mixed code and document component variation in exposure timing and CDI definitions. | Exposure assessed at diagnosis, index admission, or within the prior 90 days; outcomes used symptoms plus toxin, toxin only, or ICD-9 codes. | Component-level heterogeneity supported retaining the aggregate/mixed classification rather than assigning one study-level code to the pooled estimate. |
| Anjewierden component audit | 14 pediatric studies overall; 4 in the PPI pool | Retain the aggregate/mixed code and identify the actual PPI evidence subset. | The PPI OR derived from 4 adjusted studies; CDI testing and timing varied. | The full 10.5-million denominator was not attributed to the PPI contrast. |
| Floria component audit | 8 PPI-versus-placebo RCTs | Retain the aggregate/mixed code and document follow-up and event variation. | 60/14,946 versus 49/14,934 events; follow-up ranged from days or hospital stay to approximately 3 years. | Low event accrual and variable follow-up supported treating the pooled estimate as aggregate/mixed evidence without reclassifying the pooled RR. |
| Higher-NOS original-report restriction | Chang 2020 and Li 2022 excluded | Restrict the eight original observational reports to those with NOS scores ≥7, as reported in Supplementary Table S1. | Six reports remained: Moreels 2024, Jiang 2025, Lin 2022, Boven 2026, Huang 2024, and Boustany 2023. | Heterogeneity in outcome targets, temporal alignment, and ascertainment remained; the interpretation did not depend solely on the two lower-NOS reports. |

Overall interpretation: Across the rule-based restrictions, methodological heterogeneity persisted. None of these analyses established that outcome observability caused the direction or statistical significance of an effect estimate; they were used only to assess the dependence of the descriptive interpretation on eligibility, overlapping data sources, outcome definition, temporal alignment, ascertainment, aggregate evidence, and study quality.

**Supplementary Table S3. Independent reviewer assignments, consensus classifications, and inter-reviewer agreement.**

Panel A. Original independent assignments and consensus classifications. Each domain cell is presented as R1/R2/C.

| **Evidence source** | **Target outcome and risk set** | **PPI exposure contrast** | **Temporal alignment** | **Outcome ascertainment** |
| --- | --- | --- | --- | --- |
| Moreels 2024 | E/E/E | E/E/E | E/P/E | P/P/P |
| Jiang 2025 | P/P/P | U/P/U | U/U/U | P/P/P |
| Lin 2022 | E/E/E | P/P/P | P/E/P | E/E/E |
| Boven 2026 | E/E/E | E/E/E | E/P/E | P/P/P |
| Huang 2024 | E/E/E | E/E/E | E/E/E | P/P/P |
| Arriola 2016 | A/E/A | A/E/A | A/E/A | A/P/A |
| Anjewierden 2019 | A/A/A | A/A/A | A/A/A | A/A/A |
| Chang 2020 | E/E/E | E/E/E | E/E/E | E/E/E |
| Li 2022 | E/E/E | E/E/E | E/E/E | E/E/E |
| Boustany 2023 | E/E/E | U/U/U | U/U/U | P/P/P |
| Floria 2025 | A/A/A | A/A/A | A/A/A | A/A/A |

Abbreviations: R1, Reviewer 1; R2, Reviewer 2; C, consensus; E, explicit; P, partial; U, unclear or insufficiently reported; A, aggregate or mixed evidence for which a single uniform study-level classification was not assignable. The R1 and R2 entries reproduce the original independent assignments, whereas the C entry records the final consensus classification after discussion.

Supplementary Table S3 (continued). Independent reviewer assignments, consensus classifications, and inter-reviewer agreement.

Panel B. Domain-specific raw agreement and unweighted Cohen's kappa.

| **Analysis set** | **Domain** | **Agreements** | **Assignments** | **Raw agreement** | **Unweighted Cohen's kappa** |
| --- | --- | --- | --- | --- | --- |
| All 11 evidence sources | All domain assignments | 36 | 44 | 81.8% | Not calculated |
| All 11 evidence sources | Target outcome and risk set | 10 | 11 | 90.9% | 0.81 |
| All 11 evidence sources | PPI exposure contrast | 9 | 11 | 81.8% | 0.73 |
| All 11 evidence sources | Temporal alignment | 7 | 11 | 63.6% | 0.48 |
| All 11 evidence sources | Outcome ascertainment | 10 | 11 | 90.9% | 0.86 |
| Eight original reports | All domain assignments | 28 | 32 | 87.5% | Not calculated |
| Eight original reports | Target outcome and risk set | 8 | 8 | 100.0% | 1.00 |
| Eight original reports | PPI exposure contrast | 7 | 8 | 87.5% | 0.77 |
| Eight original reports | Temporal alignment | 5 | 8 | 62.5% | 0.37 |
| Eight original reports | Outcome ascertainment | 8 | 8 | 100.0% | 1.00 |

Raw agreement was calculated as the number of identical independent assignments divided by the total number of assignments. Cohen's kappa was calculated separately for each domain using the nominal E, P, U, and A categories without weighting. An overall kappa was not calculated across domains because the domains represent different constructs and the aggregate/mixed category was structurally applicable to meta-analytic reports. The original-report analysis excluded Arriola 2016, Anjewierden 2019, and Floria 2025.

Because only 11 evidence sources, including eight original reports, were assessed, the kappa estimates should be interpreted descriptively and not as precise estimates of framework reliability. Agreement among the eight original reports provides the more direct assessment of reproducibility for study-level coding.

Supplementary Table S3 (continued). Independent reviewer assignments, consensus classifications, and inter-reviewer agreement.

Panel C. Independent coding disagreements and rule-based consensus resolution. Study-specific evidence supporting the final classifications is summarized in Tables 2A and 2B of the main manuscript.

| **Evidence source** | **Domain** | **R1** | **R2** | **Final code** | **Rule-based consensus resolution** |
| --- | --- | --- | --- | --- | --- |
| Moreels 2024 | Temporal alignment | E | P | E | The index date and exposure windows relative to the index date were explicitly defined. |
| Jiang 2025 | PPI exposure contrast | U | P | U | PPI exposure was reported, but a clearly anchored pre-specimen or pre-outcome exposure window could not be established. |
| Lin 2022 | Temporal alignment | P | E | P | CDI cohort entry and the 180-day mortality risk period were explicit, but temporal handling of PPI exposure in relation to the secondary outcome was incompletely specified. |
| Boven 2026 | Temporal alignment | E | P | E | The case-control index date and the 30-day pre-index exposure window were explicitly defined. |
| Arriola 2016 | All four domains | A/A/A/A | E/E/E/P | A/A/A/A | The meta-analysis combined 23 heterogeneous component studies; therefore, no single uniform study-level classification was assigned to the pooled estimate. |

Note: Arriola 2016 contributed four domain-level disagreements; therefore, the five rows in Panel C represent eight domain-level disagreements in total.
